# Bone Morphogenetic Proteins and Diabetic Retinopathy

**DOI:** 10.3390/biom11040593

**Published:** 2021-04-18

**Authors:** Khaled Elmasry, Samar Habib, Mohamed Moustafa, Mohamed Al-Shabrawey

**Affiliations:** 1 Department of Cellular Biology and Anatomy, Medical College of Georgia, Augusta University, Augusta, GA 30912, USA; elmasryanatomy@gmail.com; 2 Culver Vision discovery Institute, Augusta University, Augusta, GA 30912, USA; mmoustafa@augusta.edu; 3 Department of Anatomy, Mansoura Faculty of Medicine, Mansoura University, Dakahlia Governorate 35516, Egypt; 4 Department of Medical Parasitology, Mansoura Faculty of Medicine, Mansoura University, Dakahlia Governorate 35516, Egypt; parasitologist2012@gmail.com; 5 Department of Obstetrics and Gynecology, Medical College of Georgia, Augusta University, Augusta, GA 30912, USA; 6 Department of Oral Biology and Diagnostic Sciences, Dental College of Georgia, Augusta University, Augusta, GA 30912, USA

**Keywords:** bone morphognetic proteins, BMP2, BMP4, diabetic retinopathy, age-related macular degeneration

## Abstract

Bone morphogenetic proteins (BMPs) play an important role in bone formation and repair. Recent studies underscored their essential role in the normal development of several organs and vascular homeostasis in health and diseases. Elevated levels of BMPs have been linked to the development of cardiovascular complications of diabetes mellitus. However, their particular role in the pathogenesis of microvascular dysfunction associated with diabetic retinopathy (DR) is still under-investigated. Accumulated evidence from our and others’ studies suggests the involvement of BMP signaling in retinal inflammation, hyperpermeability and pathological neovascularization in DR and age-related macular degeneration (AMD). Therefore, targeting BMP signaling in diabetes is proposed as a potential therapeutic strategy to halt the development of microvascular dysfunction in retinal diseases, particularly in DR. The goal of this review article is to discuss the biological functions of BMPs, their underlying mechanisms and their potential role in the pathogenesis of DR in particular.

## 1. Introduction

Diabetic retinopathy (DR) is one of the most common vascular complications of diabetes mellitus. The diagnosis of DR relies on the identification of microvascular abnormalities which are characterized by the breakdown of the blood retinal barrier (BRB), microaneurysms and pathological retinal neovascularization (RNV). The early stage of DR is called non-proliferative DR (NPDR), in which patients suffer from diabetic macular edema (DME) due to vascular hyperpermeability. DME is the most common cause of vision loss in diabetic patients due to the swelling and thickening of the macula that results from vascular leakage. The late stage of the disease is called proliferative DR (PDR) due to the development of RNV [1,2]. The goals of the current therapeutic strategies for DR are to prevent the inflammatory response, stabilize the BRB and prevent RNV. These goals have been achieved through laser photocoagulation and intravitreal injection of anti-vascular endothelial growth factor (VEGF) agents, corticosteroids or both. Although anti-VEGF therapy is the current mainstay for the treatment of DR and significantly improves vision with less ocular side effects, the Diabetic Retinopathy Clinical Research Network (DRCR.net) study (Protocol I) reported that two years of anti-VEGF treatment showed ≥3-line improvement in best-corrected visual acuity (BCVA) in only ~29% of DME patients [3]. This modest response to anti-VEGF suggests that DME is multifactorial and the involvement of signaling pathways other than VEGF during the development of DR.

Intravitreal sustained release corticosteroid devices such as triamcinolone acetonide, dexamethasone and fluocinolone acetonide are beneficial, especially in patients with insufficient response to anti-VEGF, serving as anti-inflammatory agents and VEGF inhibitors [4,5]. They were reported to stabilize retinal capillaries, and hence prevent the leakage of plasma proteins into the retinal tissue [1]. Dexamethasone intravitreal implants have gained importance particularly in patients with persistent DME, although their effects were shown to be time-limited [6]. Unfortunately, increased cataract-related side effects in phakic eyes, conjunctival hemorrhage, elevated intra-ocular pressure (IOP) and ocular pain were reported [7,8,9]. Thus, uncovering other pathways that may contribute to the pathogenesis of microvascular dysfunction in DR is still of great importance and may provide novel therapeutic targets to develop new alternative treatments that may overcome the limitations of current therapies [10].

The retina is a highly specialized neuronal tissue which has the ability to convert visible light into an electrochemical signal. This signal is carried to the brain, which interprets it as vision. BRB plays a fundamental role in maintaining the privilege of the eye by the regulation of the fluid and molecular movement between ocular vascular beds and retinal tissues. The BRB includes the inner blood retinal barrier (iBRB) and the outer blood retinal barrier (oBRB). iBRB consists of tight junctions between retinal endothelial cells, pericytes and glial cells. On the other hand, retinal pigment epithelium (RPE) cells linked by tight junctions create the oBRB and rest on the underlying Bruch’s membrane. oBRB plays an essential role in maintaining the microenvironment of the outer retina, while iBRB maintains the microenvironment in the inner neural retina, and any dysfunction in iBRB or oBRB contributes to the pathophysiology of a number of retinal diseases, such as DR and AMD [11,12,13].

The goal of the current review article is to present BMPs and their downstream signaling systems as potential players and novel therapeutic targets to prevent, or at least minimize, the microvascular dysfunction associated with DR.

## 2. BMP Signaling Pathways

BMPs are a subgroup of the transforming growth factor β (TGFβ) superfamily. There are ~20 BMPs which share structural similarities and were first described as key players in the formation and repair of bones (Urist, 1965, Urist and Strates, 1971). BMP family members are classified according to their structural homology into numerous subgroups, including the BMP2/4 group, the BMP5/6/7/8 group, the BMP9/10 group and the BMP12/13/14 group. Different BMPs are produced as inactive large pre-pro-polypeptides (Katagiri and Watabe, 2016). The inactively synthesized BMPs contain mature polypeptides at their carboxyl terminals, with pro-domains separating them from signal peptides at their amino terminals (Xiao et al., 2007). Mature BMPs include seven cysteines, where one of them is included in the process of dimerization by a covalent disulfide bond with another BMP monomer, generating a BMP-dimer capable of binding to, and activating, a BMP receptor (Bragdon et al., 2011). BMPs have other biological functions beyond their role in bone formation and repair. For example, BMP2 is crucial for retinal development, and both BMP2 and BMP4 are implicated in the formation of cardiac septa, and their deficiency may cause heart defects in mice. Moreover, BMP7 plays a role in the development of the kidney and the heart [14,15,16]. Recently, there is a growing interest in exploring the biological functions of BMPs and their potential roles in the pathophysiology of several diseases. 

**Figure 1:**
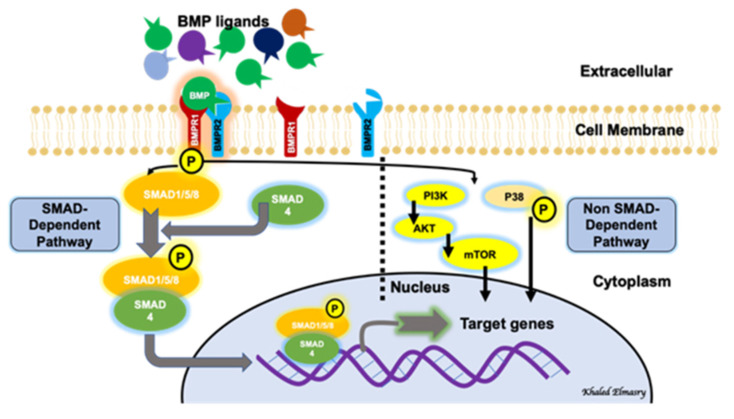
BMP signaling pathway involves both a canonical (SMAD-dependent) and a non-canonical (non-SMAD-dependent) pathway.

BMP signaling involves a canonical pathway and a non-canonical one (Figure 1). The canonical pathway is a SMAD-dependent pathway, while the non-canonical pathway involves the activation of other non-SMAD-dependent intracellular pathways, such as MAPK, IP3/AKT [17,18,19]. This reflects the complexity of the BMP-signaling pathway and indicates the presence of possible multi-levels of regulation of this significant biological axis. BMPs are initially generated as precursor protein dimers in the cytoplasm, then cleaved to form N- and C-terminal fragments. The C-terminal fragment is the one that can bind to its receptor non-covalently. In the canonical pathway, BMPs initiate a signal transduction cascade via the formation of a hetero-tetrameric complex after binding to cell surface receptors. The complex consists of two dimers of type I and type II serine/threonine kinase receptors [20]. The mechanism of action of the heterotetrameric complex is different with different types of BMPs. Regarding BMP2 and BMP4, these molecules bind type I receptors and then recruit type II receptors to form the complex. After the formation of the receptor-complex, a type II receptor, which is constitutively active, activates a type I receptor through trans-phosphorylation. The phosphorylated type I receptor then phosphorylates downstream substrate proteins called receptor-regulated SMADs [21], including SMAD1, SMAD5 and SMAD9 (SMAD1/5/9). SMAD1/5/9 then binds to SMAD4. The nuclear translocation of the SMAD1/5/9/4 complex, which acts as a transcription factor, results in multiple downstream target genes’ expression regulation. The non-canonical pathway of BMP signaling involves the intracellular activation of the MAPK pathway, the PI3/AKT pathway, Rho-GTPases and others [18].

## 3. BMP Receptors

Studies showed that BMPs are capable of binding to two types of serine-threonine kinase BMP receptors (BMPR1 and BMPR2) [20,22]. BMPs have a higher affinity for BMPR1 than BMPR2. BMPR2 is constitutively active even in ligand absence [23]. Both receptors are structurally similar and consist of an intracellular domain with serine-threonine kinase activity, a single transmembrane domain and a short extracellular domain. BMPs can also bind to activin type II receptors ACVR2A and ACVR2B [24], which are expressed in different tissues. BMP receptors can be classified structurally into different subgroups. BMP type I receptors can be subdivided into the activin receptor-like kinase 3 (ALK3, or BMPR-IA)/ALK6 (BMPR-IB) group and the ALK1/ALK2 group. ALK2 and ALK3/6 are broadly expressed in many cell types, while ALK1 expression is mainly limited to endothelial cells [25]. BMP type II receptors (BMPR2) include BMPR-II, which is specific for BMPs, and ActR-II and ActR-IIB, which are shared by activins and myostatin [26]. Other studies identified multiple BMP co-receptors, by which BMP ligands/receptors interactions are modified. There are two co-receptors playing principal functions in vascular development and disease, including endoglin and betaglycan [27], through which BMP signaling can be activated [28,29]. Proliferating endothelial cells expressing endoglin as a transmembrane protein are capable of binding to multiple ligands, including BMP-2/7 [30].

## 4. BMP Signaling Regulation and Endothelial Cell Function

It was believed that the extracellular matrix (ECM) represented an inert mechanical barrier getting rid of BMPs. However, recent work demonstrated that ECM may have a role in regulating BMP signaling [31]. BMP signaling is regulated at different and multiple layers. Interestingly, the existence of inhibitory SMADs (I-SMADs), which are members of the SMAD family, plays an important regulatory function not only on BMP-signaling pathways, but also on the TGF-β superfamily regulation. I-SMADs include SMAD6 and SMAD7. Moreover, SMADs 1/5/9 are the receptor-mediated SMADs (R-SMADs), via which BMP signaling is mediated, while SMAD4 is a co-SMAD that shares in the formation of the active complex which will be translocated to the nucleus to act as a transcription factor for multiple downstream target genes. I-SMADs have conserved carboxy-terminal MH2 domains, that interact with both activated type I receptors and R-SMADs inhibiting BMP-intracellular signaling. SMAD6 inhibits the SMAD-dependent signaling pathway mediated via BMP type I receptors ALK-3 and ALK-6 [32], while SMAD7 inhibits both TGF-β- and BMP-mediated SMAD signaling pathways [33]. 

BMPs promote angiogenesis by facilitating endothelial motility and invasion, as well as cell proliferation [34]. During embryonic and postnatal retinal angiogenesis, a crosstalk between BMP-SMAD and Notch signaling is necessary for stalk cell specification in the endothelium [35]. Moreover, both in vitro and in vivo studies have shown that BMP2 and BMP4 mediate pro-angiogenic effects through VEGF-A/VEGFR2 and angiopoietin-1/TIE2 signaling stimulation [35,36]. BMP signal transduction is involved in the regulation of physiological as well as pathological processes of the endothelium. BMP signaling has been shown to be involved during multiple pathological conditions where vascular hyperpermeability is a typical hallmark, such as acute inflammation, atherosclerosis and metastasis [35]. However, the precise role of BMP signaling in endothelial cell permeability control remains elusive.

The interplay between BMPs and immune response should be taken into consideration. Studies on macrophages and endothelial cells have suggested both pro-inflammatory and anti-inflammatory roles [37,38]. Tumor necrosis factor (TNF)-α was found to induce the expression of BMP2 in human umbilical vein endothelial cells (HUVECs) [39] and in chondrocytes via NFκB [40], suggesting a pro-inflammatory role of BMP2.

Monocytes from type 2 diabetic patients express higher levels of BMP2. Additionally, human macrophages shift to the M1 inflammatory phenotype when exposed to high glucose. Accordingly, higher levels of BMP2 in type 2 diabetes may contribute to the activation of the inflammatory response [41,42].

Several studies have highlighted the role of BMP2 in endothelial cell inflammation [43,44,45]. Pardali et al. [39] used primary human monocytes to prove that BMP2 is a potent monocyte chemoattractant, where PI3K, P38 and MAPK are involved in signaling. They reported that BMP2 hinders macrophages’ differentiation to the M2 phenotype, which is responsible for the resolution of inflammation and healing. Moreover, they reported that BMP2 increases the adhesive properties of monocytes and endothelial cells by enhancing the expression of adhesive (ICAM-1 and VCAM-1) and pro-inflammatory (IL-1β, IL-6 and IL-8) molecules. As BMP members may exert pro-inflammatory or anti-inflammatory functions, any imbalance between these members may disturb the inflammatory response [46].

Agonists and antagonists of the TGF-β family, including BMPs, were extensively reviewed previously [47]. Among this extensive network of regulators, we would like to focus on the BMP endothelial cell precursor-derived regulator (BMPER) because it is relevant to endothelial cell function. BMPER plays an essential role in the fine-tuning of BMP activity in angiogenesis in a dose-dependent manner. BMPER was shown to have a proangiogenic effect in endothelial cells that is mediated by the activation of the fibroblast growth factor (FGF) signaling [48], and demethylation reduced BMPER expression in fibroblasts [49]. Low concentrations of BMPER promote the migration of endothelial cells, while high doses do the opposite, indicating that BMPER regulates the migration of endothelial cells in a dose-dependent manner [50]. An interesting study pointed to the role of BMPER and BMP signaling in endothelial barrier function regulation. In this study, heterozygous *Bmper* knockout mice were used where there was a significantly higher vascular leakage into interstitial lung tissue when compared with wild-type mice. Moreover, *Bmper* knockdown in endothelial cells increased endothelial permeability and reduced the VE-cadherin expression. These effects were rescued by the use of the recombinant human BMPER protein. Interestingly, enhanced BMP activity induced the effects of *Bmper* knockdown on both VE-cadherin expression and endothelial permeability. Increased levels of BMPER antagonized BMP4 signaling and prevented BMP4-induced endothelial barrier dysfunction and VE-cadherin downregulation, proposing BMPER as a BMP antagonist capable of restoring the endothelial barrier function [51]. Retinas from *Bmper*^+/−^ mice showed increased vascular branching and sprouting. Although BMP levels did not increase in the retinas of those mice, BMP signaling was activated, as demonstrated by the increased endothelial phosphorylated SMAD protein levels and the increased expression of BMP target genes [52]. However, some studies showed that BMPER can be a BMP agonist, while other studies showed it as a BMP antagonist. An interesting study by Kelly et al. [53] postulated that BMPER switches from BMP4 activator to inhibitor when its molar concentrations exceed that of BMP4. The discrepancy among studies that examine how BMPER regulates BMP activity can be explained by a dosage-dependent molecular switch involving the BMPER-mediated internalization of BMP4. Interestingly, our recent study demonstrated a significant downregulation of the BMPER gene in cultured human retinal endothelial cells that were subjected to a high glucose treatment [54]. Taken together, these studies underscored the important role played by BMP signaling in endothelial cell function.

## 5. BMPs and Retinal Development

BMPs are essentially known for their salient role in embryogenesis and development. “BMPs are essentially known for their critical role in embryogenesis and development. Therefore, genetic elimination of BMPs, their downstream effectors or their receptors is embryonically lethal or causes major developmental anomalies”, their downstream effectors or their receptors were either embryonically lethal or suffered from major developmental anomalies. This reflects their striking function during the process of development. For instance, knocking out BMP 2 in mouse models showed that it is embryonically lethal, with defects in the formation of the amnion and chorion, impaired limb and cartilage development and impaired cardiac development. Animals lacking BMP 4 died in utero with a deficient development of the mesoderm and multiple organ anomalies, including cardiovascular and gonadal anomalies [55,56,57,58]. Intriguingly, conditionally knocking out BMPR-IA (ALK3) in mouse eyes showed that it is mandatory for both the lens and the retina development [59]. Moreover, it was shown that signaling through BMP4/Smad1/5/9 promotes the formation of Müller glia-derived progenitor cells in the developing retina [60], and the neural retina is specified from the forebrain cells via BMP activity [61]. A reprogramming of the neural retina during the development into retinal pigment epithelial cells was induced by BMP signaling [62]. Early postnatal retinal vascular development was investigated by using a mouse model with inducible, endothelium-specific deleted SMAD 1 and SMAD 5, where arterial-venous malformations were observed. These findings underscored the significant functions of BMP-SMAD1/5 signaling during vessel formation and remodeling in the early postnatal retinal development [63]. Interestingly, the mutation of the gene responsible for BMPR1B resulted in reduced expression levels of pSMAD1/5/9 and a defective optic nerve development, irregular retinal vessels and a defective ventral retinal development, with a reduced formation of retinal ganglion cells [64]. During embryogenesis, even the exact timing of activation of the BMP pathway is critical for the fate of the specific development, as was shown during the early differentiation of photoreceptors [65]. Moreover, endothelial cells are expressing an intracellular BMP inhibitor, SMAD6, which decreases both the sprouting and branching of the blood vessels. The genetic deletion of SMAD6 in mouse models resulted in the lethality of the mice either late in gestation or early on in the postnatal period, which was associated with vascular hemorrhage [66]. Mouse models of inducible, endothelial-specific ALK1or SMAD4 deletion suffered from arteriovenous malformations via a PI3K-dependent mechanism [67,68]. Utilizing a genetic approach, animal studies using an endothelial-specific deletion of the BMPR2, Alk1, Alk2 and Alk3 in the retinal vasculature showed reduced vascular sprouting and branching postnatally, indicating the necessity of these molecules as retinal proangiogenic effectors [69].

## 6. BMP and Diabetes-Induced Vascular Complications

BMPs are multifunctional growth factors that contribute to the pathogenesis of vascular complications of diabetes. BMPs play an important role in glucose homeostasis, being implicated in the development of the pancreas and insulin secretion [70,71]. There is a positive correlation between elevated BMPs and diabetic retinopathy [72], coronary artery diseases [73,74], atherosclerosis and vascular calcification [44]. Endothelial cells have been shown to respond readily to high glucose treatment with upregulation of BMPs and their inhibitors and receptors. This has been correlated with the increase in the levels of VEGF and inflammatory markers [44]. Experimental diabetes, such as Ins2^Akita/+^ and db/db mice, also demonstrated an increased vascular expression of BMP ligands, inhibitors and receptors [44]. Recently, we showed significant increases in the plasma levels of BMP2 in Ins2^Akita/+^ and db/db mice [54]. Moreover, enhanced BMP inhibition limited diabetic vascular disease in Ins2A^kita/+^ that lacked the BMP inhibitor matrix Gla protein (MGPtg/wt; Ins2^Akita/+^). Taken together, these data suggest the emerging role of BMPs in vascular changes during diabetes. They also suggest that the BMP signaling system could be a therapeutic target to halt the development of the vascular complications of diabetes.

## 7. BMP and Diabetic Retinopathy

Earlier studies in our lab on human pre-osteoblasts drew our attention to the important role of BMPs as regulatory players in the process of inflammation and angiogenesis via upregulating VEGF and IL-6 [75]. These previous studies led us to further investigate the role of BMPs in DR, in which inflammation and angiogenesis are cardinal pathological features [10].

We published the first report showing increased levels of retinal BMP2 in human and experimental DR [72]. In this study, BMP2 levels were increased in HRECs also subjected to BMP2. The same study showed that BMP2 disrupted the endothelial barrier function, upregulated retinal VEGF levels, induced endothelial-leukocyte adhesion and upregulated inflammatory markers and cytokines such as ICAM-1, IL-6 and IL-8. However, the mechanisms by which BMP2 induces all these changes were not investigated in this study. We recently extended our research to delineate the underlying mechanism. Our new studies detected that BMP2 induces retinal endothelial barrier dysfunction and the subsequent hyper permeability in DR via mechanisms that involve the activation of both SMAD (canonical) and non-SMAD (non-canonical) BMP signaling pathways, with the subsequent induction of VEGF, oxidative stress and inflammatory responses [54].

Microvascular endothelial cells challenged with hypoxia or treated with VEGF showed upregulated BMP2 at both RNA and protein levels [76], suggesting an important cross-talk between VEGF and BMP2 in inducing endothelial cell dysfunction associated with hypoxia or the upregulation of VEGF. Of note, hypoxia and VEGF are crucial elements of retinal hyperpermeability and RNV in DR [77,78,79]. Our previous studies established the involvement of the BMP2 signaling system in mediating retinal endothelial cell dysfunction under hyperglycemia and consistent with other studies which demonstrated the presence of a similar cross-talk between VEGF and BMP2 in HRECs [54,72]. For example, we previously showed that BMP2 levels were significantly increased in retinal samples obtained from diabetic patients and experimental diabetic mice where BMP2 expression was primarily localized in the retinal vasculature. Moreover, the secreted BMP2 levels were significantly higher in conditioned media of HRECs treated with high glucose (HG). Another group of experiments aiming to study the pro-inflammatory role of BMP2 using different concentrations demonstrated that BMP2 induced endothelial/leukocyte adhesion and upregulated HRECs’ adhesion molecule ICAM-1. Interestingly, when HRECs were treated with low BMP2 concentrations, both anti- and pro-inflammatory cytokines were significantly upregulated. Meanwhile, when HRECs were treated with higher BMP2 concentrations, only pro-inflammatory cytokines such as IL-6 and IL-8 were upregulated. BMP2-induced VEGF secretion after being added to cultured Müller cells, which represent the major cellular source of retinal VEGF. Treating HRECs with BMP2 induced retinal barrier dysfunction measured by a FITC-Dextran flux assay and Electric Cell-substrate Impedance Sensing (ECIS) [54,72].

Recently, our group reported that circulating levels of BMP2 significantly increased in plasma derived from different experimental diabetic animal models [54]. The same study showed that BMP2 also upregulates BMP receptors, ALK3 and BMPRII (BMPR2) in endothelial cells. HRECs treated with BMP2 showed upregulated mRNA levels of BMP2 and TGFβ, which may suggest the presence of a link between elevated circulating BMP2 levels in diabetes and retinal BMP2 upregulation. Moreover, a nuclear extract of HRECs treated with BMP2 showed a significant increase in nuclear p-SMAD1/5/9 and SMAD4 levels. BMPER, a known BMP2 negative regulator, was significantly reduced in HRECs treated with HG conditioned media, which may represent a possible explanation for BMP2 upregulation in diabetic retinas. Furthermore, HRECs treated with BMP2 had a significant increase in the nuclear translocation of NFκB and in phosphorylated p38 MAPK. Interestingly, the BMP2-induced disruption of the trans-endothelial resistance (TER) of HRECs was restored by inhibiting BMPRs, inhibiting p38 or silencing the SMAD pathway. Moreover, hyperglycemia-induced endothelial barrier dysfunction was reversed by using BMP inhibitors. Oxidative stress is highly implicated in the pathogenesis of DR. It was reported that BMP2 increased eNOS mRNA and reactive oxygen species (ROS) generation in HRECs, which was inhibited by using BMPR inhibitors [54]. Taken together, our data provide a strong rationale for the involvement of the BMP2 signaling system in the development of microvascular dysfunction in DR. In addition, the inhibition of BMP2 alone or in conjunction with anti-VEGF therapy could be a new therapeutic approach to treat DR.

**Figure 2:**
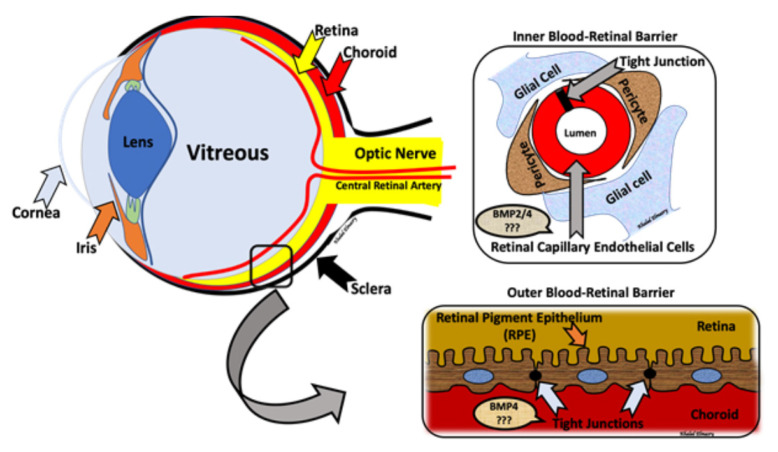
The blood-retinal barrier (BRB) is formed by an inner blood retinal barrier (iBRB) and an outer blood retinal barrier (oBRB). The inner barrier is maintained via tight junctions among retinal non-fenestrated endothelial cells. The outer blood-retinal barrier is maintained via tight junctions among retinal pigment epithelial cells (RPE). Different BMPs contribute to retinal barrier dysfunction. Our studies show the BMP2 affecting the iBRB, while changes in circulating BMP4 levels contributed to the oBRB dysfunction.

Collectively, we are suggesting the presence of a differential impact of BMPs affecting retinal-blood barriers, with BMP2 dysregulation affecting mostly the inner barrier [54,72], and circulating BMP4 dysregulation affecting mostly the outer barrier [80] (Figure 2). Retinal levels of BMP4 were detected by RNase protection assays in both non-diabetic RPE from human donors and ARPE-19 cells. Moreover, ARPE-19 cells subjected to diabetic condition showed a significant increase in secreted BMP4 levels in the conditioned media. Additionally, ARPE-19 cells treated with BMP4 showed a significant increase in VEGF secreted into the conditioned media, suggesting that BMP4 may contribute to ocular angiogenesis via a VEGF-induced mechanism [81]. Moreover, BMPs may be implicated in the pathogenesis of proliferative vitreoretinopathy (PVR), in which epiretinal contractile fibrotic membranes are formed and cause retinal detachment. The colocalization of BMP4 and its receptors was reported within the PVR membrane along with α-SMA positive cells. Interestingly, our previous studies showed a significant increase in the levels of BMP2 in vitreous samples from patients with PVR compared to other retinal diseases such as PDR [72]. In primary RPE cells, BMP4 inhibited TGF-β-induced EMT, cell migration and Smad2/3 phosphorylation [82]. Alk1 signaling was impaired with hyperglycemia, as noted in both cultured endothelial cells and a STZ-induced diabetic animal model. Furthermore, diabetic heterozygous ALK1 knockout mice showed retinal microvascular barrier dysfunction with increased vascular leakage. Intriguingly, ALK1 ligand BMP9, that was intravitreally delivered via adenovirus, was able to improve the retinal barrier function. This was in part via the induction of retinal occludin expression and inhibiting the VEGF-induced phosphorylation of VE-cadherin [83].

Interestingly, Xu et al. [84] have shown a negative correlation between BMP4 and TNF in mice with laser-induced choroidal neovascularization (CNV). Further, they reported that TNFα down-regulates BMP4 expression in cultured human fetal RPE cells, ARPE-19 cells and RPE cells in murine posterior eye cup explants. Another remarkable study was also conducted by Xu et al. [85], who identified the anti-angiogenic role of BMP-4 using transgenic mice over-expressing BMP4 in RPE and laser-induced CNV. Interestingly, they found that mice with BMP4 over-expression displayed a less severe CNV, and no increase in VEGF or matrix metalloproteinase (MMP)-9 levels after laser injury when compared to the control mice. Furthermore, they demonstrated that the pre-treatment of RPE cells with BMP4 in vitro reduced the TNFα-mediated MMP-9 secretion, in a SMAD-dependent mechanism. Contrary to previous studies by Xu et al. [84,85] in which BMP4 reduced the laser-induced choroidal neovascularization and TNFα-induced inflammatory response in RPE, our data suggested that BMP4 disrupts the RPE and REC barrier function and may play a role in the development of DR and AMD via activating MMPs. Although this contradiction requires further investigation, it could be attributed to the fact that our studies focused more on the direct effect of BMP4 on the RPE function, or that we used a different RPE cell line.

Remarkably, human ocular samples from neovascular age-related macular degeneration (AMD) donors were rich in BMP2 and BMP4, particularly in RPE and choroid rather than in retina. However, only BMP4 was upregulated in blood samples from those donors. Therefore, our group suggested that circulating BMP4 but not BMP2 or local retinal BMP4 is implicated in the pathogenesis of neovascular AMD. This conclusion was confirmed by demonstrating that BMP4 but not BMP2 interrupted the RPE barrier function, induced RPE migration and increased MMPs’ activity in RPE [80].

In summary, although the role of the BMP signaling system in the pathogenesis of diabetic retinopathy is under-investigated, accumulated evidence from our lab and other studies underscored the potential involvement of BMPs in inducing microvascular dysfunction in DR. Thus, targeting BMP signaling may represent a future therapeutic intervention to treat DR.

## Data Availability

Not applicable.
